# Supplementary material for: Violence in Healthcare Workers Is Associated with Disordered Eating
Source: Int J Environ Res Public Health. 2025 Aug 5;22(8):1221. doi: 10.3390/ijerph22081221 (PMC12386381; doi:10.3390/ijerph22081221)
Supplement: Supplementary file 1 [file ijerph-22-01221-s001.zip › ijerph-3787700-supplementary.pdf]

Supplement

Table S1. Moderation analysis. Relationship between WV and EDE-QS score, moderated by Work-related stress.

Variables[1]	Coefficient	p
Constant	2.75	<0.001
Any WV	2.06	<0.05
Work-related stress (ERI)	2.05	<0.001
Interaction	-0.61	0.43

[1] Outcome variable: EDE-QS score.
